# Phenotype and animal domestication: A study of dental variation between domestic, wild, captive, hybrid and insular *Sus scrofa*

**DOI:** 10.1186/s12862-014-0269-x

**Published:** 2015-02-04

**Authors:** Allowen Evin, Keith Dobney, Renate Schafberg, Joseph Owen, Una Strand Vidarsdottir, Greger Larson, Thomas Cucchi

**Affiliations:** Department of Archaeology, University of Aberdeen, St. Mary’s Building, Elphinstone Road, Aberdeen, UK; CNRS-Muséum National d’Histoire Naturelle, UMR 7209, Archéozoologie, Archéobotanique : Sociétés, Pratiques et Environnement, 55 rue Buffon, 75005 Paris, France; Group Animal Breeding, Institute of Agricultural and Nutritional Sciences (IANS), Martin-Luther-University Halle-Wittenberg, Theodor-Lieser-Str, 11 D-06120, Halle/Saale, Germany; Department of Archaeology, Simon Fraser University, Education Bulding 9635, 8888 University Dr, Burnaby, BC V5A Canada; Department of Anthropology, Durham University, South Road, Durham, DH1 3LE UK; Durham Evolution and Ancient DNA, Department of Archaeology, Durham University, South Road, Durham, DH1 3LE UK

**Keywords:** Teeth, Molars, Geometric morphometrics, Biogeography, Artificial selection, Natural selection

## Abstract

**Background:**

Identifying the phenotypic responses to domestication remains a long-standing and important question for researchers studying its early history. The great diversity in domestic animals and plants that exists today bears testament to the profound changes that domestication has induced in their ancestral wild forms over the last millennia. Domestication is a complex evolutionary process in which wild organisms are moved to new anthropogenic environments. Although modern genetics are significantly improving our understanding of domestication and breed formation, little is still known about the associated morphological changes linked to the process itself. In order to explore phenotypic variation induced by different levels of human control, we analysed the diversity of dental size, shape and allometry in modern free-living and captive wild, wild x domestic hybrid, domestic and insular *Sus scrofa* populations.

**Results:**

We show that domestication has created completely new dental phenotypes not found in wild boar (although the amount of variation amongst domestic pigs does not exceed that found in the wild). Wild boar tooth shape also appears to be biogeographically structured, likely the result of post-glacial recolonisation history. Furthermore, distinct dental phenotypes were also observed among domestic breeds, probably the result of differing types and intensity of past and present husbandry practices. Captivity also appears to impact tooth shape. Wild x domestic hybrids possess second molars that are strictly intermediate in shape between wild boar and domestic pigs (third molars, however, showing greater shape similarity with wild boar) while their size is more similar to domestic pigs. The dental phenotypes of insular *Sus scrofa* populations found on Corsica and Sardinia today (originally introduced by Neolithic settlers to the islands) can be explained either by feralization of the original introduced domestic swine or that the founding population maintained a wild boar phenotype through time.

**Conclusions:**

Domestication has driven significant phenotypic diversification in *Sus scrofa*. Captivity (environmental control), hybridization (genome admixture), and introduction to islands all correspond to differing levels of human control and may be considered different stages of the domestication process. The relatively well-known genetic evolutionary history of pigs shows a similar complexity at the phenotypic level.

**Electronic supplementary material:**

The online version of this article (doi:10.1186/s12862-014-0269-x) contains supplementary material, which is available to authorized users.

## Background

Understanding the evolutionary mechanisms involved in the process of domestication provides crucial insights into how wild animals and plants have been shaped over time by varying degrees of human intervention and control. Experiments and studies on domesticated animals have long been of interest to evolutionary biologists and indeed played a pivotal role in Darwin’s initial development of the theory of natural selection (e.g. [1]). Darwin contrasted the process of artificial selection by humans with that of natural selection in the wild [2] and, in doing so, highlighted the general evolutionary mechanisms that led to past and present phenotypic diversity between wild and domestic organisms. Thus, for the last 150 years, distinguishing between the phenotypic responses brought about by artificial selection induced by domestication from those due to natural selection in the wild has been a major challenge for both evolutionary biologists and archaeologists alike.

With its wild ancestral form (*Sus scrofa* Linnaeus, 1758) widely distributed throughout Eurasia, wild boar is an excellent taxa in which to study the geographic origins of its domestication and subsequent dispersal with early farmers. Whilst numerous genetic studies have greatly improved our understanding of such fundamentally important questions (e.g. [3-5]), little, however, is known about the related morphological changes involved in the domestication process beyond the well-studied phenomenon of size reduction [6].

The complex nature of domestication means that there is not a simple dichotomy between wild and domestic forms (e.g. [7]). For the purpose of animal domestication studies, five distinct categories have been described: i.e. wild, captive wild, domestic, cross-breeds and feral [8,9].

**Wild** forms are primarily subject to natural selection, although the action of past demographic events and artificial selection induced by game management or habitat destruction cannot be excluded. In the West Palearctic, wild boar display significant variation in size and shape (e.g. [6,10]), which has led to the description of several sub-species [11,12].

**Captive wild** animals are directly affected by a relaxation of natural selection associated with feeding, breeding and protection/confinement by humans, and an intensification of artificial selection through passive selection for animals that are more suited to captivity [13]. This category provides a unique opportunity to assess the relative importance of environmental and genetic factors upon morphology [13].

**Domestic** animals are mainly subject to intensified artificial selection through diverse husbandry practices (both in the past and present), with relaxation of natural selection associated with captivity and management. A great variety of domestic pig breeds exist today [14] that possess a diversity of phenotypic traits. Although domestic pigs may be affected to some degree by the same environmental factors as wild boar (climate, food availability, etc.), selection is mainly influenced by local husbandry practices, ranging from free-range extensive management of regionally specific varieties [15] to complex breeding schemes and confinement in modern-day industrialised units.

**Cross**-**breeds** are genetic hybrids of wild and domestic parents. They provide useful information on the mode of inheritance of traits, since hybrids may either be ‘forms’ intermediate between both parents, forms more similar to one parent than the other, or unique forms distinct from both parents [16,17]. Hybrids can be intentionally bred for hunting purposes [18] or for the production of meat with specific characteristics [15], whereas unintentional hybrids can be the result of contact with wild individuals when domestic pigs are reared in free-range conditions (e.g. [19,20]). Although hybridization between wild and domestic pigs occurs at low frequency under modern husbandry regimes [21], it likely played a more significant and continuous role in the history of animal domestication than has been previously considered [22,23].

**Feral** animals are domesticates that have returned to a wild state. As such, they experience relaxed artificial and (at the same time) natural selection induced by the captive environment, paired with intensified natural selection induced by the wild habitat [9]. Pigs have been introduced successfully to many areas of the world, including many islands and in many of these cases, both the timing of introduction and their wild-domestic status have often been unclear [24]. Extant wild populations of Corsican and Sardinian are well known examples of this process. Their introduction by early Neolithic settlers is a more likely scenario than them representing a post glacial relic population [25-28]. However, the question remains as to the status of specimens when introduced – i.e. as either domestic or wild forms. This uncertainty is also further complicated by an ‘island effect’ which, has likely induced peculiar morphologies and size change, both of which can mimic the process of domestication (e.g. [29,30]).

Direct evidence of animal domestication in the archaeological record has in the past essentially involved attempts to establish a simple wild - domestic dichotomy, principally through changes in the size of bones and teeth [31]. The likely occurrence of continued introgression between these two forms [22] - and the existence of a range of other forms intermediate between the wild and domestic ‘extremes’ (see above) - means that more refined methods of exploring phenotypic changes are needed to fully document and understand the complexity of domestication as seen through the zooarchaeological record [32-36].

Here we present a morphometric study of dental variation in 502 modern *Sus scrofa* specimens. We focus specifically on size, shape and allometry in the five basic forms that encompass the wild-domestic range. Biogeographic patterns in true wild boar populations are explored in order to describe the impact of ‘natural’ environment on tooth size and shape, whilst historic domestic breeds are used to assess the heterogeneity of human control. After describing the differences between the two extremes (wild/domestic) of the domestication process, the overall phenotypic variation is explored by including captive, hybrid and feral-insular *Sus scrofa* populations (the latter deriving from the Mediterranean islands of Sardinia and Corsica). This approach allows us to explore the true complexity and multiformity of the domestication process in West Palaearctic pigs, at least at the level of dental phenotype.

## Results

**Figure 1:**
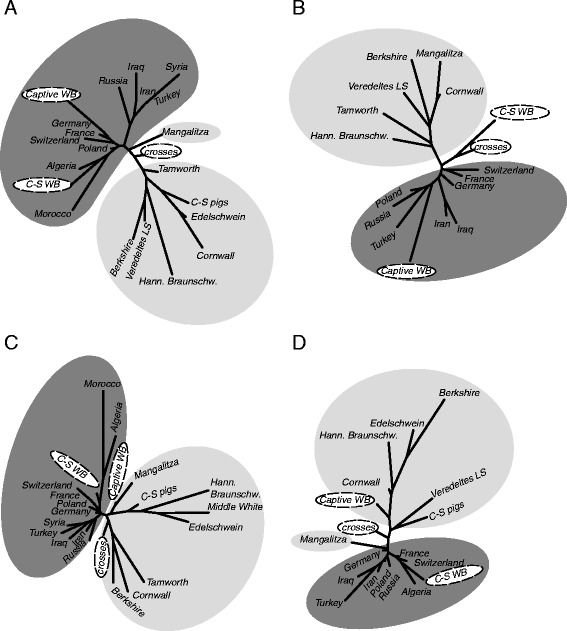
**Neighbor**-**joining networks displaying overall molar shape similarities.** Neighbor-joining networks of D^2^-Mahalanobis distances displaying overall molar shape similarities between domestic breeds (light grey), feral/insular pigs (white), captive wild boar (white), wild and domestic crosses (white) and wild boar from various geographic origins (dark grey) for M^2^ **(A)**, M^3^ **(B)**, M_2_ **(C)** and M_3_ **(D)**. (LS: Landschwein, Hann. Braunschw.: Hannover Braunschweiger Landschwein, C-S WB : Corsican and Sardinian wild boar, C-S pigs: Corsican and Sardinian domestic breeds).

In this study we analysed a total of 1204 molars (both upper and lower 2^nd^ and 3^rd^ molars [M^2^, M^3^ and M_2_, M_3_ respectively]) from the five categories outlined previously: wild, domestic, captive wild, hybrid, as well as insular populations from Corsica and Sardinia. Molar size and shape variation and covariation (allometry) were analysed using 2 dimensional landmarks and sliding semi-landmarks based on geometric morphometric approaches (SI-Figure 1).

### Overall phenotypic variation

**Figure 2:**
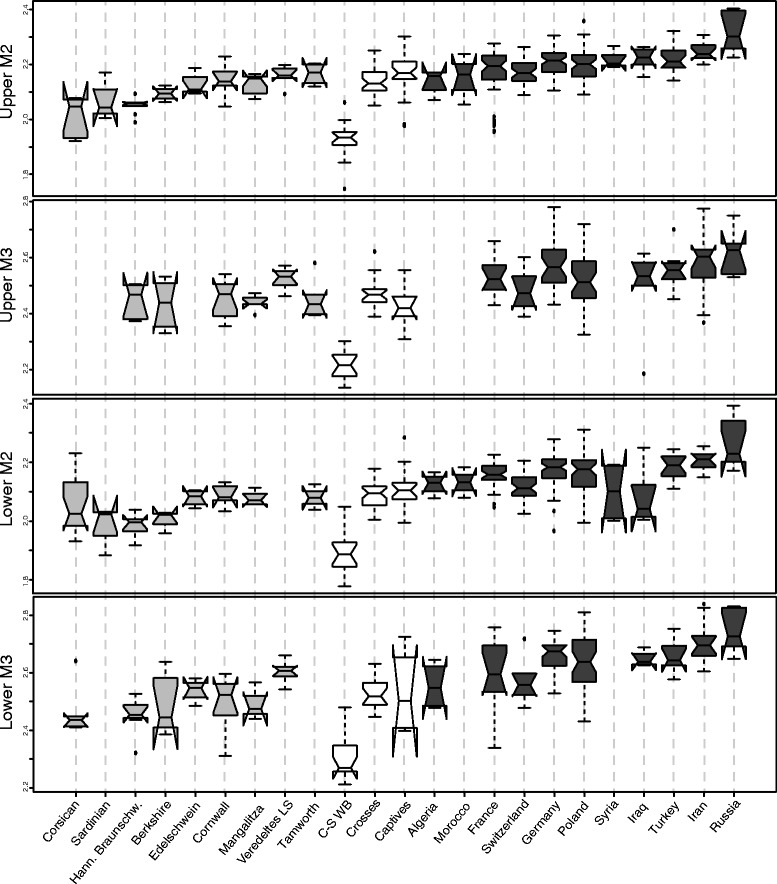
**Overall molar size variation.** Overall molar size (log-transformed centroid size) variation among domestic breeds (light grey), insular wild boar, captive wild boar, crosses between wild and domestic pigs (all in white) and wild boar from various geographic origins (dark grey).

All four teeth studied show a clear structuring of phenotypic variation for both shape (Figure 1) and size (Figure 2, Additional file 1: Figure S1) (all Kruskal Wallis’s test and MANOVA with p < <0.05, pairwise comparisons are depicted in Additional file 2: Tables S1-S4). With the exception of the domestic Mangalitza breed - which interestingly shows greater molar shape affinity with wild boar for M^2^ (Figure 1A), and M_3_ (Figure 1D), and to some extent for M_2_ (Figure 1C), all of the domestic breeds (and those pigs from Corsica and Sardinia) cluster on one side of the shape networks (Figure 1). Whilst wild boar populations cluster on the opposite side of the networks, they further divide into two subgroups - clearly identifiable for the second molars (Figure 1A, C), but not as clear for third molars (Figure 1B, D). One group comprises specimens from the Near-East (Iraq, Iran, Syria and Turkey) and Russia, the others being from Europe (Poland, Switzerland, Germany, France) and North-Africa (Morocco and Algeria).

**Figure 3:**
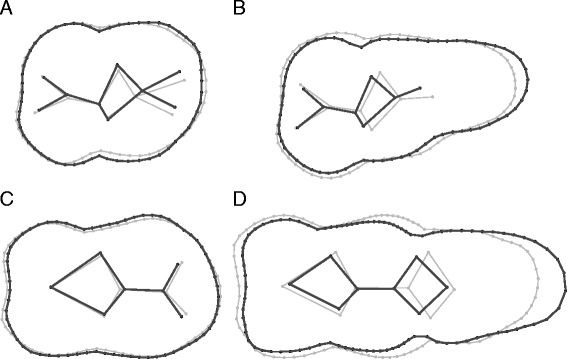
**Visualization of allometry in wild boar.** Visualization of allometry in wild boar. Large specimens are represented in black, small specimens in grey. Visualizations along size axes for M^2^ **(A** **)**, M^3^ **(B** **)**, M_2_ **(C** **)** and M_3_ **(D** **)**.

Our data appear to show wild boar molar size, shape and allometry to be strongly affected by geographic location (Table 1, see Additional file 2: Tables S1-S4 for pairwise comparisons). Western European specimens are smaller than those from Eastern Europe (Figure 2). Russian specimens have the largest teeth and North African specimens (Algeria and Morocco) the smallest (Figure 2). Allometries for M^2^, M^3^ and M_3_ all differ by geographic location (Table 1). In wild boar, it appears that between 30% and 80% of the total variance in shape is explained by variance in size (adjusted R^2^: M^2^: 0.3, M^3^: 0.4, M_2_: 0.8, M_3_: 0.5, all p < 0.001), with allometric differences especially visible in third molars (Figure 3B and D). Here large specimens show proportionally longer talonids (narrowing throughout the tooth length) and internal cusps partially shifted forward (Figure 3).

**Table 1:** **Size**, **shape and allometry comparisons for wild and domestic pigs**

		**M** ^**2**^		**M** ^**3**^		**M**_**2**_		**M**_**3**_	
		**Statistic**	***p***	**Statistic**	***p***	**Statistic**	***p***	**Statistic**	***p***
Wild	Size	**X2** **(** **10** **) =** **45.41**	**2e**-**6**	**X2** **(** **7** **) =** **17.13**	**0.02**	**X2** **(** **10** **)** **=** **58.33**	**7 e**-**9**	**X2** **(** **8** **) =** **29.80**	**2e** **-** **4**
	Shape	**F** **((** **210** **,** **2460** **) =** **2.72**	**<** **2e** **-** **16**	**F** **(** **147** **,** **707** **) =** **2.28**	**1 e** **-** **12**	**F** **(** **50** **,** **1235** **) =** **2.26**	**<** **2e**-**16**	**F** **(** **128** **,** **896** **) =** **2.02**	**4 e** **-** **9**
	Allometry	**F** **(** **210** **,** **2350** **) =** **1.41**	**2e**-**4**	**F** **(** **147** **,** **651** **) =** **1.24**	**0.04**	F(360, 2100) = 1.03	0.34	**F** **(** **128** **,** **824** **) =** **1.37**	**6 e** **-** **3**
Domestic	Size	**X2** **(** **9** **) =** **34.64**	**7 e**-**5**	X2(5) = 10.90	0.05	**X2** **(** **8** **) =** **20.85**	**0.008**	**X2** **(** **6** **) =** **21.27**	**1 e** **-** **3**
	Shape	**F** **(** **90** **,** **432** **) =** **2.67**	**2e** **-** **11**	**F** **(** **55** **,** **140** **) =** **1.75**	**0.005**	**F** **(** **56** **,** **308** **) =** **1.88**	**4 e** **-** **4**	**F** **(** **54** **,** **192** **) =** **2.46**	**4 e** **-** **6**
	Allometry	F(90, 342) = 1.06	0.36	-	-	F(56, 245) = 0.81	0.83	F(54, 150) = 0.89	0.69
Wild/Domestic	Size	**W** **=** **2257**	**<** **2e** **-** **16**	**W** **=** **1097**	**1 e** **-** **7**	**W** **=** **1395**	**<** **2e** **-** **16**	**W** **=** **702**	**6 e** **-** **13**
	Shape	**F** **(** **29** **,** **297** **) =** **11.08**	**<** **2e**-**16**	**F** **(** **25** **,** **137** **) =** **11.16**	**<** **2e** **-** **16**	**F** **(** **15** **,** **295** **) =** **17.34**	**<** **2e** **-** **16**	**F** **(** **20** **,** **150** **) =** **14.08**	**<** **2e** **-** **16**
	Allometry	**F** **(** **29** **,** **295** **) =** **2.03**	**1 e**-**3**	**F** **(** **25** **,** **135** **) =** **2.51**	**4 e** **-** **4**	**F** **(** **15** **,** **293** **) =** **2.09**	**0.01**	**F** **(** **20** **,** **148** **) =** **3.61**	**3 e**-**6**

Differences in size, shape and allometry among the different geographic origins of wild boar, among the different breeds of domestic pig, and between wild and domestic pigs. Results correspond to the statistic of the tests (Kruskal-Wallis (X2), Wilcoxon (W), and analysis of variances (F)) paired with the corresponding probability (p). Degrees of freedom are mentioned in brackets. Results in bold are significant after correcting for multi-test comparisons.

**Figure 4:**
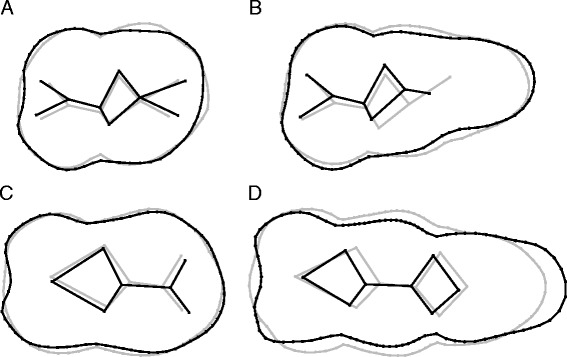
**Shape differences between domestic pigs and wild boar.** Shape differences between domestic pigs (grey) and wild boar (black). Visualizations along the CVA axes for M^2^ **(A)**, M^3^ **(B)**, M_2_ **(C)** and M_3_ **(D)**.

The domestic (historic) and insular breeds used in this study differ from each other in their molar size for all teeth except M^3^ (Table 1, Additional file 2: Tables S1-S4). The developed breeds Veredeltes Landschwein and Tamworth pigs have the largest teeth, whereas Berkshire, Corsican and Sardinian breeds have the smallest (Figure 2). Despite small sample size, all domestic breeds differ markedly in tooth shape (Table 1), whilst allometries appear homogeneous amongst breeds (Table 1). Pooling all breeds, allometries are significant for the M^2^ (adjusted R^2^ = 0.27, p = 0.004), M^3^ (adjusted R^2^ = 0.70, p = 6e-4) and M_3_ (adjusted R^2^ = 0.54, p = 4e-5), but not for M_2_ (p = 0.06).

### Differences between wild boar and domestic pigs

Because overall phenotypic variation appeared to be clearly structured by the impact of domestication (Figures 1 and 2), it was possible to study the differences between wild boar and domestic pigs in more detail. On average, wild boar have larger teeth than domestic pigs (Figure 2, Table 1) - as well as a distinctive molar shape (Table 1); wild boar possess proportionally narrower teeth - especially the third molars that also have proportionally longer talonids (Figure 4). The shape of the occlusal surface (measured by true landmarks) also differs between the two groups, with clear antero-posterior shifts observed for mandibular teeth, and lateral shifts for maxillary teeth (Figure 4). The relationship between size and shape (i.e. allometric patterns) also differs between wild boar and domestic pigs (Table 1). Within wild boar, shape differences along the allometric axis (Figure 3) appear (at least to some extent) similar to the differences observed between wild and domestic groups (Figure 4). To exclude the possibility that allometry explains most of the shape differences between the two groups, we removed its effect from shape analyses using allometry-free residuals. Taxinomic prediction of specimens using the original shape datasets yield 88.1% (M^2^), 92.5% (M^3^), 91.5% (M_2_) and 92.9% (M_3_) of correct classification (similar to those obtained in [33]), whereas only 79.7% (M^2^), 87.5% (M^3^), 82.1% (M_2_) and 77.4% (M_3_) were correctly identified using the allometry-free residuals.

In the four molars studied, we found no differences in the amount of size variation between wild and domestic pigs, whereas only three of the four teeth show no differences in molar shape variation (Table 2). The only exception is the M_3,_ for which domestic pigs are more diverse in molar shape than wild boar (Table 2) – with a mean shape distance of 0.05 for domestic pigs and 0.04 for wild boar.

**Table 2:** **Comparison of size and shape variances between wild boar and domestic pigs**

	**Size**		**Shape**	
	**Khi2**	***p***	**Khi2**	***p***
M^2^	0.039	0.84	0.23038	0.65
M^3^	2.2854	0.13	2.3544	0.12
M_2_	0.2271	0.63	3.2738	0.07
M_3_	0.2927	0.59	**8.9651**	**0.003**

The Khi^2^ values of the Fligner-Killeen test are provided along with the corresponding probability (p). Results in bold are significant after correcting for multi-test comparisons.

### Captivity and hybridization

In our study, captive wild specimens did not systematically differ in size from wild boar or domestic pigs (Table 3). Captive wild boar appear closer to domestic pigs in the size of their third molars, but conversely similar to wild boar in the size of their second molars (Figure 2, Table 4). For all teeth, captive specimens show a molar shape closer to wild boar than to domestic pigs (Figure 1, Table 4).

**Table 3:** **Pairwise comparisons of the different groups**

		**M** ^**2**^		**M** ^**3**^		**M**_**2**_		**M**_**3**_	
		**Statistic**	***p***	**Statistic**	***P***	**Statistic**	***p***	**Statistic**	***p***
Captive vs WB	Size	W = 4219	0.03	**W** = **752**	**9 e**-**4**	**W** = **3830**	**3 e**-**4**	W = 377	0.12
	Shape	**F** **(** **8** **,** **284** **) =** **6.16**	**2e**-**7**	**F** **(** **4** **,** **125** **) =** **3.18**	**0.02**	**F** **(** **13** **,** **264** **) =** **2.52**	**3 e**-**3**	F(2, 130) = 0.87	0.42
	Allometry	-	-	F(7, 120) = 0.93	0.49	F(13, 262) = 0.79	0.67	-	-
Captive vs DP	Size	**W** **=** **355**	**2e** **-** **4**	W = 186	0.18	**W** **=** **254**	**7 e** **-** **4**	W = 82	0.96
	Shape	**F** **(** **12**, **71** **) =** **11.11**	**5 e**-**12**	**F** **(** **4** **,** **42** **) =** **8.10**	**6 e** **-** **5**	**F** **(** **12** **,** **60** **) =** **3.99**	**2e**-**4**	F(3, 42) = 0.56	0.64
	Allometry	F(12, 69) = 0.70	0.75	-	-	F(12, 58) = 1.73	0.08	-	-
Crosses vs WB	Size	**W** **=** **2106**	**1 e** **-** **8**	**W** **=** **592**	**8 e** **-** **5**	**W** **=** **1419**	**5 e** **-** **11**	**W** **=** **306**	**4 e** **-** **8**
	Shape	**F** **(** **13** **,** **291** **) =** **5.00**	**6 e**-**8**	**F** **(** **7** **,** **136** **) =** **7.15**	**3 e** **-** **7**	**F** **(** **10** **,** **282** **) =** **12.16**	**<** **2e**-**16**	**F** **(** **6** **,** **142** **) =** **6.96**	**2e** **-** **6**
	Allometry	F(13, 289) = 0.98	0.47	**F** **(** **7** **,** **134** **) =** **2.53**	**0.02**	F(10, 280) = 0.71	0.71	F(6, 140) = 1.03	0.41
Crosses vs DP	Size	**W** **=** **1425**	**0.01**	W = 437	0.8	**W** **=** **1290**	**2e** **-** **3**	W = 476	0.41
	Shape	**F** **(** **21** **,** **74** **) =** **5.51**	**2e** **-** **8**	**F** **(** **7** **,** **53** **) =** **7.85**	**2e** **-** **6**	**F** **(** **9**, **78** **) =** **10.81**	**1 e**-**10**	**F** **(4** **,** **57) =** **8.63**	**2e** **-** **5**
	Allometry	F(21, 72) = 1.27	0.23	-	-	F(9, 76) = 0.95	0.49	-	-
Crosses *vs* captive	Size	**W** **=** **306**	**0.02**	W = 106	0.09	W = 266	0.15	W = 42	0.91
	Shape	**F** **(12** **,** **49) =** **9.49**	**4 e** **-** **9**	**F** **(4** **,** **23) =** **19.85**	**3 e** **-** **7**	**F** **(7** **,** **47) =** **8.93**	**5 e** **-** **7**	F(4, 19) = 1.25	0.32
	Allometry	F(12, 47) = 1.32	0.24	-	-	-	-	-	-
C-S WB vs WB	Size	**W** **=** **21**	**2e** **-** **12**	**W** **=** **0**	**3 e** **-** **10**	**W** **=** **8**	**2e** **-** **11**	**W** **=** **0**	**9 e** **-** **10**
	Shape	**F** **(8** **,** **277) =** **2.33**	**0.02**	**F** **(8** **,** **129) =** **3.75**	**5 e** **-** **4**	**F** **(8** **,** **264) =** **4.02**	**1 e** **-** **4**	**F** **(12** **,** **130) =** **8.54**	**9 e** **-** **12**
	Allometry	F(8, 275) = 1.06	0.39	**F** **(8** **,** **127) =** **2.68**	**9 e** **-** **3**	F(8, 262) = 0.37	0.94	F(12, 128) = 0.81	0.64
C-S WB vs DP	Size	**W** **=** **1024**	**3 e** **-** **9**	**W** **=** **600**	**2e** **-** **13**	**W** **=** **797**	**1 e** **-** **7**	**W** **=** **577**	**7 e** **-** **11**
	Shape	**F** **(16** **,** **60) =** **10.99**	**2e** **-** **12**	**F** **(6** **,** **48) =** **13.61**	**6 e** **-** **9**	**F** **(9** **,** **59) =** **2.64**	**0.01**	**F** **(5** **,** **50) =** **11.03**	**3 e** **-** **7**
	Allometry	F(16, 58) = 0.93	0.55	6, 46) = 1.43	0.22	F(9, 57) = 1.01	0.44	F(5, 48) = 0.68	0.64
C-S WB vs captive	Size	**W** **=** **445**	**6 e** **-** **11**	**W** **=** **105**	**1 e** **-** **5**	**W** **=** **317**	**2e** **-** **9**	**W** **=** **56**	**7 e** **-** **4**
	Shape	**F** **(11** **,** **31) =** **10.57**	**1 e** **-** **7**	**F** **(5** **,** **16) =** **4.44**	**0.01**	**F** **(6** **,** **29) =** **3.21**	**0.02**	F(4, 13) = 1.00	0.44
	Allometry	F(11, 29) = 1.73	0.12	F(5, 14) = 2.49	0.08	F(6, 27) = 0.42	0.86	F(4, 11) = 0.33	0.85
C-S WB vs crosses	Size	**W** **=** **663**	**1 e** **-** **13**	**W** **=** **315**	**4 e** **-** **10**	**W** **=** **554**	**8 e** **-** **12**	**W** **=** **280**	**1 e** **-** **9**
	Shape	**F** **(21** **,** **33) =** **9.22**	**2e** **-** **8**	**F** **(6** **,** **29) =** **8.30**	**3 e** **-** **5**	**F** **(9** **,** **41) =** **5.61**	**5 e** **-** **5**	**F** **(7** **,** **26) =** **14.37**	**2e** **-** **7**
	Allometry	F(21, 31) = 1.51	0.14	F(6, 27) = 1.37	0.26	F(9, 39) = 0.49	0.87	F(7, 24) = 0.97	0.48

Pairwise comparisons of wild boar (WB), domestic pigs (DP), captive wilds, crosses and Corsican and Sardinian wilds (C-S WB). Differences are tested using Wilcoxon tests for size and MANOVA for shape and MANCOVA for allometry. The values of the statistics (W and F) are provided along with the degrees of freedom in brackets and the corresponding probability (p). Results in bold are significant after correcting for multi-test comparisons.

**Table 4:** **Measures of proximity between groups**

		**Tooth**											
		**Upper M2**			**Upper M3**			**Lower M2**			**Lower M3**		
	**Comparison**	**Mean(D2)**	**W**	**p**	**Mean(D2)**	**W**	**p**	**Mean(D2)**	**W**	**p**	**Mean(D2)**	**W**	**p**
Shape	DP-captive WB	12.026	**10000**	**<** **2.2e**-**16**	187.591	**7672**	**6.69E** **-** **11**	8.157	**9793**	**<** **2.2e** **-** **16**	3.755	5368	0.3692
	WB-captive WB	**3.755**			**62.824**			**3.697**			3.396		
	WB-DP	7.732			115.946			8.767			8.005		
	DP-crosses	3.548	5494	0.2279	8.546	**6925**	**2.57e** **-** **06**	5.870	4657	0.4027	8.452	**6625**	**7.00e** **-** **05**
	WB-crosses	3.432			**7.299**			5.998			**7.590**		
	WB-DP	6.794			11.441			7.013			13.697		
	DP-CS WB	18.620	**8766**	**<** **2.2e** **-** **16**	11.646	**7544**	**5.14e** **-** **10**	**6.759**	**3151**	**6.28e** **-** **06**	21.918	**7587**	**2.62e** **-** **10**
	WB-CS WB	**12.503**			**8.939**			8.179			**15.811**		
	WB-DP	11.421			9.497			13.914			13.492		
Size	DP-captive WB	0.825	**9496**	**<** **2e** **-** **16**	**0.361**	**279**	**<** **2e** **-** **16**	1.072	**7567**	**3.50e** **-** **10**	**0.168**	**1031**	**<** **2e** **-** **16**
	WB-captive WB	**0.259**			2.512			**0.671**			1.080		
	WB-DP	1.917			1.231			3.361			1.650		
	DP-crosses	**0.308**	**247**	**<** **2e** **-** **16**	**0.040**	**1**	**<** **2e** **-** **16**	**0.451**	**2**	**<** **2e** **-** **16**	**0.101**	**0**	**<** **2e** **-** **16**
	WB-crosses	0.997			1.249			1.786			2.505		
	WB-DP	2.367			1.581			3.994			3.420		
	DP-CS WB	**7.606**	**100**	**<** **2e** **-** **16**	**5.989**	**0**	**<** **2e** **-** **16**	**5.989**	**0**	**<** **2e** **-** **16**	**8.685**	**103**	**<** **2e** **-** **16**
	WB-CS WB	17.103			18.187			18.187			22.951		
	WB-DP	1.987			3.402			3.402			3.543		

Mean Mahalanobis D^2^ distances between groups of interest, with results of the comparison of the distance distributions between captive WB, crosses or CS WB and the wild and domestic pigs (Wilcoxon’s tests (W) and corresponding probability (p)). The smallest distances are in bold.

**Table 5:** **Sample size by tooth and category**

		**M** ^**2**^	**M** ^**3**^	**M**_**2**_	**M**_**3**_
Wild boar	Algeria	4	0	4	4
	Morocco	4	0	3	0
	France	51	12	42	12
	Switzerland	40	8	40	9
	Germany	68	31	73	33
	Poland	52	37	52	39
	Syria	5	0	4	0
	Iraq	5	6	5	4
	Turkey	20	8	15	7
	Iran	12	15	12	14
	Russia	7	6	8	7
**Total wild boar**		**268**	**123**	**258**	**129**
**Corsican** **+** **Sardinian WB**		**5** **+** **13**	**0** **+** **15**	**5** **+** **11**	**0** **+** **14**
Domestic pigs	Corsican	6	0	17	5
	Sardinian	4	0	4	0
	Berkshire	10	6	7	7
	Hannover Braunschweiger LS	4	5	4	6
	Edelschwein	7	0	4	4
	Cornwall	6	10	5	8
	Mangalitza	11	5	6	3
	Veredeltes LS	7	9	0	9
	Tamworth	4	5	3	0
	Middle White	0	0	3	0
**Total domestic**		**59**	**40**	**53**	**42**
**Captive wild**		**25**	**7**	**20**	**4**
**Crosses** **(** **F1** **+** **F2** **)**		**8** **+** **29**	**8** **+** **13**	**7** **+** **28**	**8** **+** **12**
**Total**		**407**	**206**	**382**	**209**

Crosses are from first (F1) and second (F2) generations.

Wild-domestic crosses differ from wild boar in both size and shape in all teeth, but only differ by their allometric trajectories for the M^3^ (Table 3). Crosses also differ from domestic pigs in shape, but only differ in the size of both upper and lower second molars (Table 3). Crosses between wild and domestic pigs fall between the two parental groups in size (Figure 2), but are closer to domestic pigs (Table 4). These crosses are strictly intermediate in shape for second molars (M^2^ and M_2_), but closer to wild boar for both upper and lower third molars (Table 4).

Wild-domestic crosses and captive wild specimens differ only in the size of their M^2^s, without allometric changes, and in all molar shapes (with the exception of M_3_ (Table 3))_._

### Insular wild populations

In our study, island (Corsican and Sardinian) wild populations differ both in size and shape from mainland wild boar and domestic pigs (Table 3). Firstly, they possess significantly smaller teeth than all wild and domestic pigs in our study (Figure 2, Tables 3 and 4) - smaller even than the Corsican and Sardinian domestic breeds living on the same islands (Figure 2). The Corsican and Sardinian wild populations are closer in molar shape to mainland wild boar (with the exception of the M_2_ [Table 4]) and interestingly show especially close shape similarities with North-African wild boar (Figure 1A and D). No differences were detected in allometric patterns between these island groups and wild boar or domestic pigs - with the exception of M^3^ (Table 3). The Corsican and Sardinian wild populations also show significant molar size and shape differences to both the captive wild boar (with the exception of M_3_ shape) and wild-domestic hybrids, although all groups show similar allometric patterns for (Table 3).

## Discussion

The purpose of our study was to assess *Sus* phenotype diversification (measured by molar size, shape and allometry) in different populations involved (or not) to varying degrees in pig domestication. We specifically explored evolutionary outcomes – i.e. through detailed differences in dental (molar) morphology within and between populations with distinct evolutionary histories – but obviously did not directly study the actual process of selection itself.

### Domestication: size, shape and allometry

Our data clearly demonstrate that domestication induces strong morphological changes in both molar size and shape. Since the domestic pig genome shows a strong signature of selection [4] - but no strong founder effect [37], artificial selection can be considered the principal evolutionary force acting upon animal domesticates. In order to attain certain desired characteristics or use for specific roles, humans have imposed strong selective pressures over millennia on the ancestral gene pool of animals during the domestication process [9]. For pigs these selective pressures have primarily been for meat production, involving faster gestation and larger litters, as well as rapid and larger muscle growth [38-40].

Domestication is well known to have induced an overall reduction in body size in many species [41] and domestic pigs are no exception - having, on average, smaller molars than wild boar, although there is a significant overlap in size [10,33,42]. In addition to molar size differences, wild and domestic pigs also appear to differ in molar shape - with proportionally narrower (and occasionally longer) teeth found in wild boar. The well-documented shortening of the face and dental row in domestic animals [2] is also reflected in longitudinal compression of the molars (especially the third molars), and has been previously described for domestic pig populations from Island South East Asia [36,43].

In addition to differences in molar size and shape, wild boar and domestic pigs also differ strongly in their allometric profiles. Shape variation associated with allometric trajectories in wild boar appears (to some extent) comparable to the shape differences noted between wild and domestic pigs. This hints at a link between allometry and domestication - a pattern previously shown for dog breed diversification [44,45]. When the common allometric component is removed from the analysis of shape, differences between wild and domestic pigs remain, with only a marginal increase in overlap (<10%). This suggests that allometry alone cannot be invoked to distinguish wild pigs from their domestic counterparts, and that both size and shape are affected by artificial selection during domestication.

Irrespective of differences in the local, natural and human environment that they inhabit, domestic breeds share common morphometric characteristics (in terms of molar size and shape) compared to wild boar. Domestication appears to have favoured completely new dental phenotypes not found in the wild. However, the amount of variation within wild boar and domestic pigs appears similar for three of the four teeth studied. This result is congruent with the absence of a strong founder effect during domestication and with similar amounts of genetic divergence occurring between wild and domestic pigs as has been observed between various European wild boar populations [37]. Only for the lower third molar was the variation among domestic pigs slightly higher than that observed in the wild.

A previous study of domestic dog and wolf crania reported that the amount of shape variation among domestic dogs greatly exceeded that found in the wild [45]. Three possible explanations have been purported for these results: Firstly, dogs were domesticated for many distinct purposes, such as herding, guarding, hunting, rescuing, or companionship [46], resulting in the large number of very specialised modern breeds. Secondly, teeth and skulls are likely not subject to the same selective natural or anthropogenic pressures - both elements not necessarily being the target of selection but selected through genetic correlation, developmental and/or functional constraints [47]. Thirdly, teeth and crania likely do not have the same ecophenotypic plasticity - skulls being genetically and functionally more complex than teeth [47].

In the same way, different constraints appear to act on second and third molars - something that has previously been observed during the history of pig domestication [6,48,49]. Second molars are constrained by their position between the first and third molars, whereas the third molar is only constrained on its anterior side, which may explain its greater variation. Of relevance here is a study on non domestic murine rodents that has demonstrated an increasing variance from the first to the third molar potentially due to genotypic, developmental and functional constraints [50].

### Biogeographic patterns under natural selection

Wild boar populations show clear patterns of biogeographic variation in both molar size and shape. At least sixteen sub-species of *Sus scrofa* are currently recognized, based primarily on their external morphologies [11,12] - with support from genetic markers [27]. Our results reveal two main groups of Eurasian wild boar based on molar shape. The first includes Eastern West-Eurasian populations (Turkey, Syria, Iran, Iraq and Russia) that likely correspond to a mix of *S. scrofa scrofa*, *S. scrofa attila* and *S. scrofa lybicus*. The second includes Western specimens that can be further divided into two additional sub-groups: those of North African origin (Morocco and Algeria) that correspond to the proposed subspecies *S. scrofa algira*, and those of European origin (France, Germany, Poland, Switzerland) that correspond to the nominal subspecies *S. scrofa scrofa* [12]. This East-West clinal phenotypic differentiation is concordant with the recently established pattern of genetic variation purporting to reflect different glacial refugia [51].

Eastern populations have much larger molars than western populations – supporting a previously well documented East-West cline for decreasing body size in many animals including *Sus scrofa* [6,11,12,52]. Bergmann’s rule ([53] - later reformulated [54]) - predicts a relationship between body size and climate, where one should expect larger body size linked to colder climatic conditions. Several evolutionary factors may explain this relationship, including thermoregulatory mechanisms, latitudinal differences in primary productivity or differences in environmental predictability (review in [55]). *Sus scrofa* clearly complies with the principles of Bergmann’s rule [55], not only in terms of dental measurements ([56]; this study) but also in overall morphology. This is also manifested by increased hair cover, a shorter and stockier body, shorter tail and smaller ears [57] generally all linked with colder climates.

### Breed diversity

The several hundred domestic pig breeds officially recognized today [14] vary significantly in their external morphology, reflecting differing local environmental factors, husbandry practices and selection strategies. Despite their complex genetic heritage, each breed used in this study – and known to differ in overall body size (e.g. [58]) - displays a unique combination of molar size and shape. One breed in particular (the traditional Hungarian landrace Mangalitza) is the only breed that shares a specific mutation with wild boar at the MC1R coat color locus, and the only one of 51 breeds studied whose piglets are striped like wild boar piglets [59]. Interestingly, in our study, the Mangalitza shares some similarity in molar shape with wild boar, showing that the phenotypic proximity of Mangalitza to wild boar is not limited to the expression of coat color, but also extends to the dental phenotype. This could indicate either less intensive artificial selection, or the introgression of wild boar into the domestic gene pool at some recent point in the breed’s history.

### Differential selection and dental phenotypes

‘Wild’ and ‘domestic’ are the two extremes of a domestication continuum [7]. Whilst artificial selection is an important force acting on the ancestral gene pool and shaping the domestic phenotype, other populations (e. g captive wild boar; crosses between wild boar and domestic pigs) represent different stages along the continuum. Artificial selection induces changes through two possible evolutionary mechanisms: a) conscious selection through selective breeding based on target characters; or b) unconscious selection through isolation within a controlled human environment [60]. Captive wild boar that are subjected to a relaxation of natural selection should theoretically also undergo morphological changes due to their captive conditions (review in [13]), even in the absence of conscious selection.

In our study, captive specimens are clearly distinct in terms of their molar shape, but are closer to wild boar than to domestic pigs in this respect. This implies that although the environment influences molar shape development, genetic background retains a strong influence over the phenotype. In contrast, captivity appears to induce a decrease in molar size to such an extent that the size of some captive specimens teeth becomes similar to that of domestic pigs, highlighting the strong effect of local environment on size. The majority of captive wild boar used in this study represent specimens from the historic livestock garden of the Martin-Luther-University of Halle-Wittenberg (Germany), where they had been captive for at least three generations. Additionally, we cannot rule out that the captive patterns observed here are influenced by inbreeding effects, which of course would be expected in small captive populations [9,61].

Wild boar and domestic pigs have been sympatric over most of their range and could have hybridized over millennia – and most likely did [22,23]. Today the estimated contribution of hybrids to wild boar populations is very low (<5%) [21], although this could have been very different in the past. Since they represent a direct genetic admixture of both wild and domestic backgrounds, hybrids do allow us to study the heritability of phenotypic characters. Hybrid phenotypes may not necessarily be intermediate between the two parents - the result of complex epistatic interactions between differentiated genomes (see references in [62]). The teeth of crossbreeds included in this study are relatively small - closer in size to domestic pigs, and not significantly different to the captive wild boar, highlighting the importance of environmental and genetic factors associated with captivity in bringing about size reduction observed during domestication. For shape, however, crosses show strong differences to all other groups (except for M_3_ comparison with captive wild boar) and are strictly intermediate in shape between wild boar and domestic pigs for all molars except M^2^. This is consistent with the hypothesis that hybrid molar shape is at least partly controlled by a cumulative effect of the two parental genomes - a result that can only be confirmed by further study of hybrids of known parental origin.

### The ‘wild’ *Sus* populations of Corsica and Sardinia

Feralisation is another important mechanism in the domestication trajectory. The wild populations of Corsica and Sardinia correspond to the recognised sub-species *S. scrofa meridionali*s [12] and show specific patterns of size and shape variation that can be linked with both their genetic background and insular isolation. Like the Corsican and Sardinian mouflon (*Ovis gmelinii musimon*) [63,64], the extant populations on these islands are thought to have descended from escaped domestic animals originally introduced by early Neolithic farmers [26–29; 60]. As expected from insular populations, the specimens from Corsica and Sardinia have very small molars compared to all other groups ([28], this study), while shape shows (for three of the four teeth) greater similarities with wild boar than domestic pigs. On Cyprus, true wild boar were introduced by epipalaeolithic hunter-gatherers as early as 12000 years ago, probably as a game species [65]. A similar scenario is unlikely for the Corsican and Sardinian populations, since the earliest secure occurrence of archaeological *Sus scrofa* remains in these Tyrrhenian islands is recorded in Corsica during the middle of the 7^th^ millennium BC [25]. Similar to the situation proposed for Cyprus [65], however, the small size observed in Corsican and Sardinian wild populations must (at least partially) be the result of evolution within an insular context subsequent to their introduction. Morphological evolution of mammals on islands can occur very rapidly [66] - some small mammals have become larger and large animals have become dwarfed (i.e. [29,67,68]), following the so-called island rule [69]. The small size of wild boar on islands (i.e. this study, [70,71]) could be the result of a similar phenomenon. Conversely, the recent Corsican and Sardinian domestic pigs are larger than the ‘wild’ populations from the same islands and, therefore, appear less affected by insularity. To what extent insular free-range breeds and wild boar living in the same habitat share the same selective pressures remains a matter of conjecture deserving of further investigation.

The observed ‘wild’ shape signature of Corsican and Sardinian wild populations in our study can be interpreted through two separate hypotheses: 1) a return to a wild phenotype during the feralisation of introduced domestic pigs (through the actions of natural selection, founder effect and/or drift combined with a relaxation of artificial selection); or 2) the introduction of managed wild boar who maintained their wild phenotype through time. The first hypothesis implies that the domestic signature (as manifested by molar shape) could be (at least partially) a reversible process.

Interestingly, our study shows that (in three of the four teeth) from Corsican and Sardinian ‘wild’ populations, molar shape values cluster in close proximity to North African *Sus scrofa algira* from Morocco and Algeria [12]. Such phenotypic affinity between insular wild populations and North-West African wild Suids (along with the European mitochondrial signature also noted in the latter [3,27]) may indicate that extant wild *Sus* populations from North West Africa are in fact feral populations of European origin - a scenario previously suggested for North East African Egyptian wild boar [72]. Our study does not, however, include modern Italian wild boar, which possess another distinctive mtDNA haplotype previously observed in Sardinian archaeological *Sus* specimens [27].

## Conclusions

West Palaearctic *Sus scrofa* present a complex pattern of dental phenotypic variation. Despite the significant impact of domestication, the dental phenotype appears to remain strongly underpinned by the biogeographic origin of wild ancestral populations. Thus molar shape in Eurasian wild boar populations is biogeographically structured into clearly defined Western and Eastern clusters, most likely reflecting intensive selection within their most recent glacial refugia. In addition to this phenotypic structuring in the wild, strong and novel selective pressures were then applied to some wild boar during subsequent domestication, inducing additional rapid and diverse phenotypic change that resulted in novel domestic phenotypes not found in the wild populations. Domestic pigs, however, do not show greater variability in their molar shape compared with their wild counterparts, a result congruent with known genetic data. The diverse domestic pig breeds included in our analyses all show distinct dental phenotypes, most likely resulting from a combination of different selective pressures brought about by specific husbandry practices/selection processes and specific local environments. Differences between true wild and captive wild boar highlight the influence of captivity as a primary source of morphological change, which may reflect those that occurred during the initial stages of domestication. In wild x domestic hybrids, the mode of inheritance of the dental phenotype appears strictly intermediate in (terms of molar shape at least) between the two parental phenotypes (for three of the four teeth), and closer to domestic pigs in terms of size.

In moving beyond the simple wild-domestic size dichotomy traditionally used when exploring the question of early animal domestication, this study has shown clear differences in the dental phenotype of wild, domestic, captive wild and hybrid *Sus* populations. As a result, the diverse forms - found all along the wild-domestic continuum - can now be explored in more detail in the zooarchaeological record through the application of GMM techniques. However, three lines of further investigation are required to conclusively test the various hypotheses developed here. First, non-insular feral and early Neolithic insular populations are required to properly contrast the effects of insularity and feralisation. Second, more specimens with known geographic origins, history, and breeding conditions are required to fully understand the effects of captivity and inbreeding on morphotype. Finally, integration with a genetic-based approach is essential for a fuller understanding of the hybrid phenotype, especially its mode of inheritance and more generally the relationship between phenotype and genotype during domestication.

## Methods

### Specimen sampling

A total of 1204 teeth (from specimens housed in international museum collections – see Table 5 for sample sizes) belonging to 502 individuals (SI-Additional file 3: Text 1) were analyzed: 407 upper second molars (M^2^), 206 upper third molars (M^3^), 382 lower second molars (M_2_) and 209 lower third molars (M_3_) (Table 5). All specimens were adult with fully erupted molars, *minus* the specimens that were too worn for the protocol to be applied. Samples were accessed from the following institutions; the Museum für Naturkunde, Berlin; Zoologische Staatssammlung, München; Muséum National d’Histoire Naturelle, Paris; Muséum d’Histoire Naturelle, Genève; National Museum of Natural History, Washington; The Field Museum, Chicago; The American Museum of Natural History, New-York; and The Museum of Domesticated Animals of the Martin-Luther-University Halle-Wittenberg, Halle (Saale). Development of bunodont teeth of *Sus scrofa* ceases after mineralisation (Hillson, 2005) therefore, any differences observed should not be related to differences in growth once the teeth are fully erupted.

The specimens analyzed were divided into the four relevant categories - wild, domestic, captive wild, and hybrids (i.e. wild-domestic crosses - see Table 5), plus the insular populations from Corsica and Sardinia. The wild and domestic specimens are the same as those analyzed in [33,73]. These previous studies focused only on the ability of size and shape to distinguish between the wild or domestic status of specimens with the aim of identifying archaeological remains. Wild specimens used in the study are of known geographic origin (country) across the west Palaeartic, domestic specimens represent specific traditional breeds with recognized origins (Table 5). We selected Western European breeds from England (Berkshire, Tamworth, Middle White and Cornwall), Southern European breeds (Corsican and Sardinian breeds that showed no significant differences and were pooled for all the analyses), and two types of Middle European varieties: traditional German breeds, which have been influenced by English bloodlines: Deutsches Edelschwein, Hannover Braunschweiger Landschwein and Veredeltes Landschwein; and finally, the traditional Hungarian landrace Mangalitza. Specimens of crosses (F1s and F2s) between domestic and wild pigs were sourced from the ‘Museum of Domesticated Animals’ at Halle, Germany. Because molar size and shape of first and second-generation wild-domestic crosses do not differ statistically in any comparisons (all MANOVA and Wilcoxon tests p > 0.05), they were pooled for all analyses.

Captive wild specimens derive from various German institutions - the majority from ‘The Museum of Domesticated Animals’, where they were likely grown for 3 to 5 generations, or kept for much longer in captivity before being bought by the institute in 1882.

Only groups with more than two specimens were analyzed, and all specimens were adults. Data from males and females were pooled after ensuring that the results were not influenced by sexual dimorphism.

### Geometric morphometric approaches

Phenotypic variation was assessed from landmarks and sliding semi-landmarks on the second and third upper and lower molars using geometric morphometric methods, following the protocol detailed in SI-Figure 1 and [33]. Each tooth was photographed in the occlusal view using a Reflex Camera (Nikon D90) coupled with a micro lens (AF-S Micro Nikkor 60 mm). The parallax was controlled by the symmetry of the anterior cusps of the teeth, and a centimeter scale was added to the pictures. 2D landmark coordinates were digitized within the occlusal surface and sliding semi-landmarks along the outline of the curves (see [33] and SI-Figure 1) using tpsDig2 v2.16 (http://life.bio.sunysb.edu/morph/, [74]). Semi-landmarks were recorded as equidistant points along two (for M^2^, M_2_ and M^3^) or four (for M_3_) curves that were delimited by extra landmarks, later one transformed as sliding semi-landmarks (SI-Figure 1). All photographs and measurements were taken by the lead author (AE).

The specimens were superimposed using a Generalized Procrustes Analysis [75,76]) and the sliding semi-landmarks were allowed to slide along a chord drawn between the adjacent points and localized to minimize the sum of Procrustes distances between each individual and the mean shape [77-80]. The superimposition and sliding process were carried out in TPS Relw v1.49 (http://life.bio.sunysb.edu/morph/, [81]). Shape coordinates (aligned specimens) and centroid size were saved and analysed using the library “Rmorph” [82] for ‘R’ [83].

Differences in the logarithm of centroid size between groups were illustrated with boxplots, and their significance tested with Wilcoxon rank tests for two-group comparisons and Kruskal-Wallis tests when more than two groups were compared. Differences in shape were tested using one-way MANOVA, with shape as the dependent variable and group as a factor, and Canonical Variate Analyses (CVA) paired with leave-one-out cross validation. Differences along CVA axes were visualized by computation of the deformations along the factorial axes by multivariate regression [84]. Due to the relatively small number of specimens and the large number of variables, we reduced the dimensionality of the data (coordinates after superimposition) using the ordination technique of principal component analysis (PCA) (i.e. [85,86]). We then used the dimensionality reduction method proposed by [85] that recommends “select[ing] the number of retained components in each analysis so as to minimize the total cross-validated misclassification percentages” ([85], p. 152). For each analyses of variance and discriminant analyzes of shape, we therefore used the N first components of the PCA that maximize the variability between the *a priori* defined groups. As unbalanced design (differences in the number of specimen by groups) can affect cross-validation results of the CVA (e.g. [33,87]), percentages of cross validation were obtained using 1000 re-sampled datasets following [33] and correspond to the upper 95^th^ percentiles of the distribution obtained for groups of same size. The overall phenotypic similarities between groups were depicted using neighbor-joining networks (unrooted trees) computed from the Mahalanobis’ *D*^*2*^ distances [88].

Only where differences in size were found, allometry was tested using multivariate regression of shape (PCA axes) on log-transformed centroid size, and homogeneity of the allometric patterns among groups was assessed using MANCOVAs with shape as the dependent variable, log centroid size as a covariate and group as a factor. To quantify the importance of allometry during domestication we contrasted the percentages of correct cross validation calculated with shape and allometry-free residuals. Allometry-free residuals were obtained by removing the effect of log centroid size on shape (PCA axes) using a pooled within-group regression [82].

To compare the amount of variation of both size and shape within wild boar and domestic pigs we computed the distances between each specimen and the consensus of its group using respectively the Euclidian distance between the log of the centroid sizes, and between the coordinates in the tangent space (i.e. the distances between the specimen and the consensus of the group). The variance of the wild and domestic pigs for size and shape were then compared using a Fligner-Killeen test of homogeneity of variances.

Differences in size, shape, and allometry between each of the three following groups: captive wild boars, hybrids and insulars, and the two extremes of the domestication process (wild boar and domestic pig) were tested with Wilcoxon test for size, MANOVA for shape and MANCOVA for allometry. To measure the proximity of the wild and domestic forms with each of the groups of interest we used Mahalanobis D^2^ distances computed separately for log-transformed centroid size and shape. As sample size heterogeneity can affect discriminant results (e.g. [33,87]) and therefore Mahalanobis D^2^, we computed distributions of the distances from 1000 resamples of groups of same size. The smallest distances will reflect a greater proximity between the group of interest and either the wild boar or the domestic pigs. The distance distributions were compared using a Wilcoxon’s test.

Due to the high number of statistical tests performed, the significance of the *p* values was assessed after adjustment for multiple comparisons following [89]. All statistical and morphometric analyzes were performed using R v 2.13.1 [83], with the ‘ade4’ [90], ‘ape’ [91], ‘Rmorph’ [82] libraries and newly designed R functions (available upon request). Centroid size, Procruste residuals and grouping factors have been deposited in the LabArchives database (doi: 10.6070/H4ZK5DNC DOI:10.6070/H4ZK5DNC#doi, [92]).

### Availability of supporting data

The data sets supporting the results of this article are available in the LabArchives repository, [http://dx.doi.org/10.6070/H4ZK5DNC].

## Additional files

Additional file 1: Figure S1. Position of landmarks (in blue) and semi-landmarks (in red) used for M^2^ **(A)**, M^3^ **(B)**, M_2_ **(C)** and M_3_ **(D)**. Landmarks along the outline were used to define the start and end points of the curves resampled with equidistant points. Then all the landmarks and equidistant points along the outline were analysed as semi-landmarks. M^2^ were measured by 10 landmarks in the occlusal view, and 58 semi-landmarks along the outline. M^3^ was measured by 8 landmarks and 68 semi-landmarks. M_2_ were measured by 7 landmarks and 68 semi-landmarks and M_3_ was measured by 8 landmarks and 91 semi-landmarks. Additional file 2: Table S1. M^2^ size (lower triangle) and shape (upper triangle) differences between groups. The table shows p-values of pairwise Wilcoxon tests for size and MANOVA for shape. Results are provided for each tooth separately. P-values were adjusted for multiple comparisons. WB: wild boar, DP: domestic pig, Hann. Braunschw.: Hannover Braunschweiger Landschwein, LS: Landschwein. **Table S2.** M^3^ size (lower triangle) and shape (upper triangle) differences between groups. The table shows p-values of pairwise Wilcoxon tests for size and MANOVA for shape. Results are provided for each tooth separately. P-values were adjusted for multiple comparisons. WB: wild boar, DP: domestic pig, Hann. Braunschw.: Hannover Braunschweiger Landschwein, LS: Landschwein. **Table S3.** M_2_ size (lower triangle) and shape (upper triangle) differences between groups for lower M2. The table shows p-values of pairwise Wilcoxon tests for size and MANOVA for shape. Results are provided for each tooth separately. P-values were adjusted for multiple comparisons. WB: wild boar, DP: domestic pig, Hann. Braunschw.: Hannover Braunschweiger Landschwein, LS: Landschwein. **Table S4.** M_3_ size (lower triangle) and shape (upper triangle) differences between groups. The table shows p-values of pairwise Wilcoxon tests for size and MANOVA for shape. Results are provided for each tooth separately. P-values were adjusted for multiple comparisons. WB: wild boar, DP: domestic pig, Hann. Braunschw.: Hannover Braunschweiger Landschwein, LS: Landschwein. Additional file 3: Text 1. List of all specimens included in the study.
