# Supplementary figures and images for: Phenotype and animal domestication: A study of dental variation between domestic, wild, captive, hybrid and insular Sus scrofa
Source: BMC Evol Biol. 2015 Feb 4;15(1):6. doi: 10.1186/s12862-014-0269-x (PMC4328033; doi:10.1186/s12862-014-0269-x)

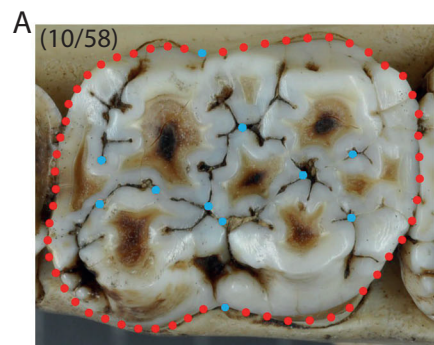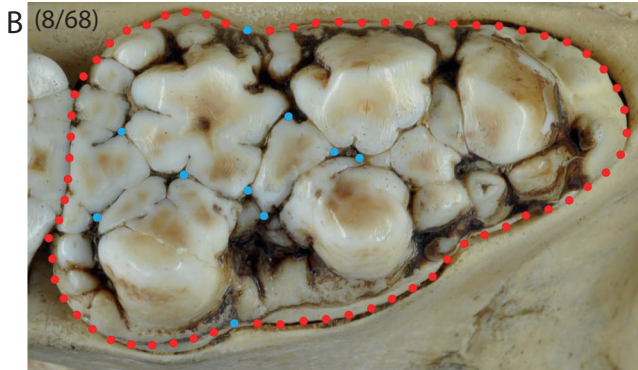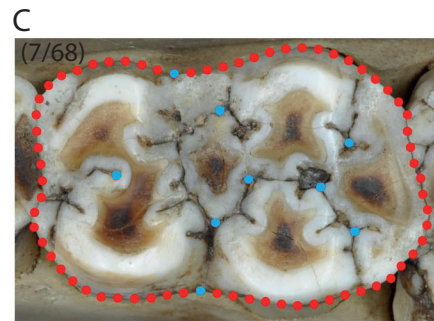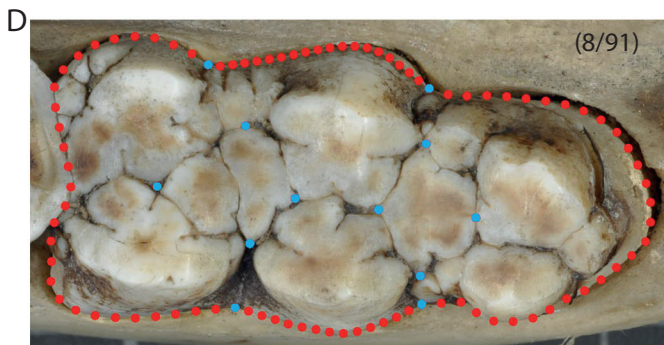

Supplement: Additional file 1: Figure S1. — Position of landmarks (in blue) and semi-landmarks (in red) used for M2 (A), M3 (B), M2 (C) and M3 (D). Landmarks along the outline were used to define the start and end points of the curves resampled with equidistant points. Then all the landmarks and equidistant points along the outline were analysed as semi-landmarks. M2 were measured by 10 landmarks in the occlusal view, and 58 semi-landmarks along the outline. M3 was measured by 8 landmarks and 68 semi-landmarks. M2 were measured by 7 landmarks and 68 semi-landmarks and M3 was measured by 8 landmarks and 91 semi-landmarks. [file 12862_2014_269_MOESM1_ESM.pdf]
