# Supplementary material for: Phenotype and animal domestication: A study of dental variation between domestic, wild, captive, hybrid and insular Sus scrofa
Source: BMC Evol Biol. 2015 Feb 4;15(1):6. doi: 10.1186/s12862-014-0269-x (PMC4328033; doi:10.1186/s12862-014-0269-x)
Supplement: Additional file 3: Text 1. — List of all specimens included in the study. [file 12862_2014_269_MOESM3_ESM.docx]

**Museum für Naturkunde, Berlin (N=160):**

nhm-16132, nhm-17606, nhm-17608, nhm-18079, nhm-18149, nhm-18178, nhm-18435, nhm-18814, nhm-20359, nhm-20756, nhm-20806, nhm-21968, nhm-22069, nhm-22070, nhm-22274, nhm-22275, nhm-22276, nhm-22277, nhm-25649, nhm-26301, nhm-26780, nhm-27204, nhm-27205, nhm-27525, nhm-29642, nhm-29643, nhm-30222, nhm-30226, nhm-30228, nhm-30229, nhm-30230, nhm-30233, nhm-30234, nhm-30235, nhm-30236, nhm-30239, nhm-30243, nhm-30246, nhm-30247, nhm-30250, nhm-30252, nhm-31054, nhm-31056, nhm-32201, nhm-32202, nhm-32338, nhm-35600, nhm-37537, nhm-37538, nhm-37539, nhm-37540, nhm-37543, nhm-40705, nhm-40707, nhm-41535, nhm-42527, nhm-44023, nhm-45405, nhm-64513, nhm-69299, nhm-69483, nhm-69485, nhm-69489, nhm-69490, nhm-69497, nhm-69498, nhm-69499, nhm-69500, nhm-69501, nhm-69502, nhm-69505, nhm-69506, nhm-69508, nhm-69512, nhm-69513, nhm-69514, nhm-69516, nhm-69531, nhm-69532, nhm-69533, nhm-69534, nhm-69535, nhm-69539, nhm-69540, nhm-69578, nhm-69579, nhm-69589, nhm-69705, nhm-69709, nhm-69722, nhm-69724, nhm-69727, nhm-69729, nhm-69731, nhm-69735, nhm-69736, nhm-69737, nhm-69738, nhm-69739, nhm-69740, nhm-69754, nhm-70202, nhm-70203, nhm-70204, nhm-70205, nhm-70206, nhm-70207, nhm-70208, nhm-70209, nhm-85988, nhm-94557, nhm-97651, nhm-97794, nhm-98090, nhm-98091, nhm-98092, nhm-98093, nhm-98098, nhm-98509, nhm-98511, nhm-98512, nhm-98513, nhm-98514, nhm-98515, nhm-98516, nhm-98517, nhm-98518, nhm-98519, nhm-64564, nhm-69511, nhm-69558, nhm-69706, nhm-69707, nhm-69730, nhm-69753, nhm-12202, nhm-24206, nhm-24876, nhm-27370, nhm-29195, nhm-29739, nhm-30221, nhm-30245, nhm-32200, nhm-69503, nhm-69507, nhm-69742, nhm-69757, nhm-98510, nhm-69486, nhm-69496, nhm-69704, nhm-69728, nhm-32337, nhm-40706, nhm-69487, nhm-69733, nhm-69741, nhm-5712, nhm-69732

**Muséum d’Histoire Naturelle, Genève (N=70):**

MHNG-1262-035, MHNG-1262-036, MHNG-1489-058, MHNG-1489-060, MHNG-1489-061, MHNG-1489-073, MHNG-1489-076, MHNG-1489-095, MHNG-1489-098, MHNG-1490-007, MHNG-1490-093, MHNG-1490-094, MHNG-1490-095, MHNG-1495-025, MHNG-1495-026, MHNG-1495-028, MHNG-1497-010, MHNG-1497-011, MHNG-1497-012, MHNG-1497-053, MHNG-1602-079, MHNG-1603-041, MHNG-1604-076, MHNG-1604-083, MHNG-1604-084, MHNG-1608-053, MHNG-1608-054, MHNG-1609-008, MHNG-1609-082, MHNG-1628-088, MHNG-1632-001, MHNG-1632-002, MHNG-1632-004, MHNG-1632-032, MHNG-1632-034, MHNG-1632-075, MHNG-2001-105, MHNG-2001-10, MHNG-2001-113, MHNG-2001-114, MHNG-2001-14, MHNG-2001-16, MHNG-2001-26, MHNG-2001-27, MHNG-2001-2, MHNG-2001-39, MHNG-2001-41, MHNG-2001-42, MHNG-2001-48, MHNG-2001-53, MHNG-2001-54, MHNG-2001-56, MHNG-2001-6, MHNG-2001-9, MHNG-761-041, MHNG-831-085, MHNG-1604-077, MHNG-765-053, MHNG-839-004, MHNG-844-020, MHNG-844-021, MHNG-844-022, MHNG-844-023, MHNG-839-003, MHNG-1606-010, MHNG-2001-1, MHNG-2001-20, MHNG-2001-44, MHNG-2001-95, MHNG-2001-97

**Zoologische Staatssammlung, München (N=23):**

zsm_1906-1377, zsm_1922-10, zsm_1950-280, zsm_1951-68, zsm_1951-355, zsm_1955-22,
zsm_1955-67, zsm_1955-232, zsm_1956-11, zsm_1956-229, zsm_1966-273, zsm_AM-1183,
zsm_AM-1185, zsm_AM-1189, zsm_1953-304, zsm_1960-69, zsm_1964-25, zsm_1925-96, zsm_1956-258, zsm_1956-261, zsm_1958-136, zsm_1966-274, zsm_AM-1190

**Muséum National d’Histoire Naturelle, Paris (N=65):**

MNHN-1876-324, MNHN-1897-302, MNHN-1897-303, MNHN-1897-307, MNHN-A2-208, MNHN-bicqueley1940, MNHN-dorrigny1943, MNHN-garvier1944, MNHN-lahaye1940, MNHN-neuilly1942, MNHN14, MNHN1912-540, MNHN1912-541, MNHN1912-542, MNHN1998-1950, MNHN26-6, MNHN39-67, MNHN-S., MNHN-S15, MNHN-S17, MNHN-S20, MNHN-S29, MNHN-S31, MNHN-S35, MNHN-S37, MNHN-S38, MNHN-S3, MNHN-S40, MNHN-S43, MNHN-S44, MNHN-S53, MNHN-S59, MNHN-S7, MNHN-SPOT933, MNHN-S13, MNHN-Cor-D-RA-09, MNHN-DPcor1, MNHN-DPsar1, MNHN-DPsar3, MNHN-DPsar6, MNHN-Cor-D-RA-08, MNHN-Cor-D-RA-10, MNHN-Cor-D-RA-11, MNHN-Cor-D-RA-17, MNHN-WBcor2, MNHN-WBsar2, MNHN-WBsar5, MNHN-Cor-WB-CDV-01, MNHN-Cor-WB-SOL-01, MNHN-Cor-WB-TRA-01, MNHN-1900-143, MNHN-1897-306,S57, MNHN-Cor-D-RA-01, MNHN-Cor-D-RA-03, MNHN-Cor-D-RA-04, MNHN-Cor-D-RA-14, MNHN-Cor-D-VI-05, MNHN-Cor-D-VI-08, MNHN-Cor-D-VI-10, MNHN-Cor-D-VI-11, MNHN-Cor-D-VI-14, MNHN-Cor-D-RA-07, MNHN-Cor-D-RA-13, MNHN-Cor-WB-CA-02, MNHN-1880-636

**The Field Museum, Chicago  (N=24):**

FMNH-47417, FMNH-57947, FMNH-97885, FMNH-98915, FMNH-42440m, FMNH-43325, FMNH-44722, FMNH-44723, FMNH-46076, FMNH-46401, FMNH-57940, FMNH-84476, FMNH-92906, FMNH-92909, FMNH-97881, FMNH-97882, FMNH-97889b, FMNH-42439, FMNH-97884, FMNH-97890, FMNH-46077, FMNH-97886, FMNH-97889a, FMNH-88690

**The American Museum of Natural History, New-York (N=11):**

148197, 42501, 69407, 69409, 69410, 69411, 85398, 88714, 34911, 88724, 88690

**National Museum of Natural History, Washington (N=6):**

194177, 233985, 283111, 327738, 476855, 341614

**The Museum of Domesticated Animals of the Martin-Luther-University Halle-Wittenberg, Halle (Saale) (N=143):**

ZNS-Safwld1, ZNS-Sbks1, ZNS-Sbks2, ZNS-Sbks6, ZNS-Sbks7, ZNS-Sbks9, ZNS-Sbks10, ZNS-Sbks35, ZNS-Sbks36, ZNS-Sbks38, ZNS-Sbks60, ZNS-Sbks65, ZNS-Sbks67, ZNS-Sbks69, ZNS-Sbks70, ZNS- Scnw1, ZNS-Scnw2, ZNS-Scnw3, ZNS-Scnw4, ZNS-Scnw5, ZNS-Scnw6, ZNS-Scnw7, ZNS-Scnw8, ZNS-Scnw9, ZNS-Scnw12, ZNS-Scnw13, ZNS-Scnw26, ZNS-Sdteds6, ZNS-Sdteds27, ZNS-Sdteds32, ZNS-Sdteds33, ZNS-Sdteds37, ZNS-Sdteds41, ZNS-Sdteds42, ZNS-Sdteds82b, ZNS-Shvbr2, ZNS-Shvbr5, ZNS-Shvbr6, ZNS-Shvbr8, ZNS-Shvbr9, ZNS-Shvbr16, ZNS-Sma2, ZNS-Sma3, ZNS-Sma5, ZNS-Sma19, ZNS-Sma22, ZNS-Sma23, ZNS-Sma29, ZNS-Sma30, ZNS-Sma38, ZNS-Sma39, ZNS-Smw4, ZNS-Smw5, ZNS-Smw6, ZNS-Stmw1, ZNS-Stmw3, ZNS-Stmw6, ZNS-Stmw7, ZNS-Stmw8, ZNS-Stmw10, ZNS-Sugr1, ZNS-Sugr2, ZNS-Sugr3, ZNS-Sugr4, ZNS-Svdlds37, ZNS-Svdlds38, ZNS-Svdlds40, ZNS-Svdlds43, ZNS-Svdlds44, ZNS-Svdlds45, ZNS-Svdlds46, ZNS-Svdlds49, ZNS-Svdlds50, ZNS-Svdlds54, ZNS-Svdlds55, ZNS-Svdlds56, ZNS-Serpwld3, ZNS-Serpwld4, ZNS-Serpwld6, ZNS-Serpwld7, ZNS-Serpwld17, ZNS-Serpwld19, ZNS-Serpwld25, ZNS-Serpwld28, ZNS-Serpwld43, ZNS-Serpwld44, ZNS-Serpwld45, ZNS-Serpwld54, ZNS-Serpwld68, ZNS-Serpwld69, ZNS-Serpwld109, ZNS-Serpwld110, ZNS-Serpwld113, ZNS-Serpwld114, ZNS-Serpwld115, ZNS-Serpwld116, ZNS-Serpwld129, ZNS-Serpwld130, ZNS-Serpwld131, ZNS-Serpwld140, ZNS-Serpwld141, ZNS-Serpwld145, ZNS-Serpwld163, ZNS-Serpwld169, ZNS-Serpwld170, ZNS-Serpwld171, ZNS-Serpwld172, ZNS-Serpwld190, ZNS-Serpwld191, ZNS-Serpwld193, ZNS-Serpwld194, ZNS-Serpwld196, ZNS-Serpwld197, ZNS-Serpwld198, ZNS-Serpwld226, ZNS-Serpwld227, ZNS-Serpwld230, ZNS-Serpwld231, ZNS-Serpwld234, ZNS-Serpwld237, ZNS-Serpwld239, ZNS-Serpwld240, ZNS-Serpwld271, ZNS-Serpwld273, ZNS-Serpwld277, ZNS-Serpwld278, ZNS-Serpwld279, ZNS-Serpwld281, ZNS-Serpwld282, ZNS-Serpwld287, ZNS-Serpwld288, ZNS-Serpwld289, ZNS-Serpwld290, ZNS-Serpwld292, ZNS-Serpwld293, ZNS-Serpwld294, ZNS-Serpwld295, ZNS-Serpwld296, ZNS-Serpwld303, ZNS-Serpwld304, ZNS-Serpwld305, ZNS-Serpwld308a, ZNS-Serpwld308b
